# Supplementary material for: Multiple environmental stressors affect predation pressure in a tropical freshwater system
Source: Commun Biol. 2024 May 29;7:663. doi: 10.1038/s42003-024-06364-6 (PMC11137014; doi:10.1038/s42003-024-06364-6)
Supplement: Supplementary file 2 — Supplementary Information [file 42003_2024_6364_MOESM2_ESM.pdf]

Supplementary Tables

Supplementary Table 1. Details of each sampling location (a. to g.). For every environmental variable, average minimum, mean and maximum values are reported, these have been recorded on 2 (for width and canopy cover) or 4 (for depth, flow, light intensity, DO, temperature, turbidity) individual sampling events at each location. Sediment type was recorded from only one sampling event.

a.

River	Site	Loc	Latitude Longitude	Values	Depth (cm)	Width (m)	Flow (m/s)	Canopy cover (%)	Light intensity (lux)	DO (mg/L)	Temperature (°C)	Turbidity (NTU)	Sediment type	Date visited
St Joseph	1	A	10.7186245 -61.4205861	Min	44.0	5.0	0.00	39.7	5631.6	13.5	24.8	1.0	Silt	April 14th & May 2nd
				Mean	47.5	5.0	0.00	44.6	16296.5	14.0	26.3	1.2		
				Max	51.0	5.0	0.00	49.4	30679.6	14.5	27.4	1.4		
St Joseph	1	B	10.7186214 -61.4206214	Min	81.0	4.0	0.00	23.8	31763.8	13.1	25.3	1.2	Rock	April 14th & May 2nd
				Mean	82.0	4.0	0.00	49.1	32938.4	13.8	26.4	1.3		
				Max	83.0	4.0	0.00	74.4	35748.2	14.5	27.6	1.3		
St Joseph	1	C	10.7187229 -61.4206169	Min	60.0	6.0	0.00	61.0	3957.4	12.1	25.0	2.0	Sand	April 14th & May 2nd
				Mean	66.5	6.0	0.00	71.1	15905.3	13.2	26.0	2.1		
				Max	73.0	6.0	0.00	81.1	29999.4	14.4	26.9	2.1		
St Joseph	2	A	10.7129085 -61.4124237	Min	50.0	2.0	0.00	67.7	307.7	8.9	24.9	0.7	Leaf litter	April 14th & May 2nd
				Mean	52.5	2.0	0.00	70.8	948.5	10.1	25.9	0.9		
				Max	55.0	2.0	0.00	73.9	2423.8	11.3	26.7	1.1		
St Joseph	2	B	10.7125659 -61.4126429	Min	69.0	4.0	0.0	65.0	294.9	7.7	24.8	1.0	Pebbles	April 14th & May 2nd
				Mean	89.0	4.0	0.0	65.6	765.7	9.4	25.7	1.1		
				Max	109.0	4.0	0.0	66.1	1426.1	11.1	26.5	1.1		
St Joseph	2	C	10.7199211 -61.4086032	Min	56.0	6.0	0.0	4.1	12078.1	7.0	26.0	0.7	Silt	April 14th & May 2nd
				Mean	59.0	7.5	0.0	18.7	25673.1	8.3	26.6	0.8		
				Max	62.0	8.0	0.0	33.4	40307.5	9.6	26.9	0.8		
St Joseph	3	A	10.6953817 -61.4069179	Min	40.0	7.0	0.0	67.7	775.1	14.6	25.1	1.8	Rock	April 23rd & May 9th
				Mean	46.5	7.0	0.0	69.2	1251.8	14.7	26.3	2.3		
				Max	53.0	7.0	0.0	70.8	2335.4	14.8	27.2	2.7		
St Joseph	3	B	10.7021109 -61.4059031	Min	45.0	7.5	0.0	49.6	724.4	12.9	25.5	0.4	Leaf litter	April 23rd & May 9th
				Mean	47.5	7.7	0.0	53.9	2943.4	13.7	26.3	0.8		
				Max	50.0	8.0	0.0	58.2	8993.1	14.6	27.1	1.1		
St Joseph	3	C	10.7027705 -61.4063281	Min	50.0	5.0	0.0	50.2	3099.2	13.9	25.6	0.3	Sand	April 23rd & May 9th
				Mean	56.5	5.0	0.0	55.5	13233.5	14.1	26.7	0.8		
				Max	63.0	5.0	0.0	60.7	41981.5	14.4	27.4	1.3		

b.

River	Site	Loc	Latitude Longitude	Values	Depth (cm)	Width (m)	Flow (m/s)	Canopy cover (%)	Light intensity (lux)	DO (mg/L)	Temperature (°C)	Turbidity (NTU)	Sediment type	Date visited
Caura	1	A	10.7092688	Min	55.0	5.0	0.3	60.2	2186.5	12.7	24.9	3.2	Leaf litter	April 21st & May 5th
			-61.3552454	Mean	56.0	5.0	0.3	63.9	9633.1	12.8	25.5	3.2		
				Max	57.0	5.0	0.4	67.5	25996.4	12.9	25.8	3.3		
		B	10.7090272	Min	53.0	7.0	0.0	59.9	3391.9	13.4	25.4	2.1	Pebbles	
			-61.3549351	Mean	56.5	7.0	0.1	79.9	24534.9	13.7	25.7	2.7		
				Max	60.0	7.0	0.3	99.8	48313.6	14.0	26.3	3.4		
		C	10.7089987	Min	52.0	2.0	0.4	67.5	3408.4	13.8	25.4	1.7	Sand	
			-61.3548328	Mean	59.0	2.0	0.7	80.6	16169.2	13.9	25.4	2.7		
				Max	66.0	2.0	0.9	93.6	44632.1	14.1	25.4	3.7		
	2	A	10.6913761	Min	73.0	10.0	0.0	56.0	2066.4	12.4	25.1	1.2	Pebbles	April 21st & May 5th
			-61.357658	Mean	74.5	10.0	0.0	63.1	5355.0	13.3	25.8	3.6		
				Max	76.0	10.0	0.1	70.1	9721.3	14.2	26.5	6.1		
		B	10.6917665	Min	63.0	7.0	0.2	27.0	6580.8	14.3	25.2	1.3	Pebbles	
			-61.3575916	Mean	64.0	7.0	0.3	37.0	12030.8	14.5	26.0	3.2		
				Max	65.0	7.0	0.4	47.0	17248.9	14.7	26.6	5.2		
		C	10.6920328	Min	53.0	8.0	0.1	57.7	871.9	14.3	25.3	0.9	Leaf litter	
			-61.3576668	Mean	57.0	8.0	0.2	58.6	5443.4	14.6	25.9	3.4		
				Max	61.0	8.0	0.3	59.6	14563.7	14.9	26.4	5.8		
	3	A	10.6807475	Min	52.0	12.0	0.2	56.9	633.5	14.1	25.1	1.4	Rock	April 20th & May 3rd
			-61.362376	Mean	54.0	12.0	0.3	58.1	4016.8	14.7	25.1	1.5		
				Max	56.0	12.0	0.3	59.2	13143.9	15.4	25.2	1.7		
		B	10.6808515	Min	60.0	9.0	0.0	57.5	745.4	13.4	24.8	1.1	Pebbles	
			-61.3624565	Mean	69.0	10.5	0.0	57.8	1392.3	14.2	25.0	1.7		
				Max	74.0	11.0	0.0	58.3	2001.1	14.6	25.1	2.2		
		C	10.6808515	Min	57.0	9.0	0.0	57.9	1509.9	13.4	24.9	1.1	Leaf litter	
			-61.3624565	Mean	62.0	9.5	0.0	58.6	3588.3	13.9	25.1	1.3		
				Max	74.0	11.0	0.0	59.1	6990.4	14.3	25.1	1.4		

c.

River	Site	Loc	Latitude Longitude	Values	Depth (cm)	Width (m)	Flow (m/s)	Canopy cover (%)	Light intensity (lux)	DO (mg/L)	Temperature (°C)	Turbidity (NTU)	Sediment type	Date visited
Lopinot	1	A	10.704669 -61.3208909	Min<br>Mean<br>Max	55.0<br>59.5<br>64.0	2.0<br>2.0<br>2.0	0.0<br>0.0<br>0.0	71.5<br>74.1<br>76.7	1296.3<br>1978.0<br>2445.4	13.2<br>13.4<br>13.7	23.9<br>25.3<br>26.6	1.3<br>1.4<br>1.5	Sand	April 19th & May 4th
		B	10.7048464 -61.3207613	Min<br>Mean<br>Max	52.0<br>52.5<br>53.0	7.0<br>7.0<br>7.0	0.0<br>0.0<br>0.0	37.1<br>41.8<br>46.5	16879.2<br>32142.3<br>44815.7	13.6<br>14.4<br>15.3	24.0<br>25.7<br>27.2	1.6<br>1.9<br>2.3	Sand	
		C	10.7054614 -61.3207949	Min<br>Mean<br>Max	64.0<br>65.0<br>66.0	4.0<br>4.0<br>4.0	0.0<br>0.1<br>0.1	54.8<br>54.8<br>54.8	4030.7<br>8483.5<br>15909.4	13.7<br>13.7<br>13.8	23.9<br>25.4<br>26.7	1.1<br>1.1<br>1.2	Pebbles	
	2	A	10.6915927 -61.3234136	Min<br>Mean<br>Max	55.0<br>57.5<br>60.0	2.0<br>2.5<br>3.0	0.0<br>0.0<br>0.0	6.2<br>46.9<br>87.7	7433.5<br>14744.4<br>23410.4	8.7<br>9.4<br>10.1	25.5<br>25.7<br>25.9	0.8<br>1.1<br>1.4	Pebbles	April 19th & May 4th
		B	10.6921781 -61.323294	Min<br>Mean<br>Max	55.0<br>57.0<br>59.0	4.0<br>4.0<br>4.0	0.0<br>0.0<br>0.0	68.8<br>69.5<br>70.1	385.2<br>1479.5<br>2392.1	9.4<br>11.5<br>13.6	25<br>25.6<br>26.1	0.8<br>1.0<br>1.2	Leaf litter	
		C	10.6924552 -61.3231855	Min<br>Mean<br>Max	53.0<br>57.5<br>62.0	9.0<br>9.0<br>9.0	0.0<br>0.0<br>0.0	51.4<br>61.1<br>70.8	2634.1<br>12112.0<br>18927.7	9.2<br>11.5<br>13.8	25.4<br>26.0<br>26.4	1.3<br>1.5<br>1.7	Silt	
	3	A	10.660151 -61.3257801	Min<br>Mean<br>Max	40.0<br>45.5<br>51.0	8.0<br>8.0<br>8.0	0.0<br>0.0<br>0.0	51.7<br>54.7<br>57.7	829.2<br>1822.3<br>3204.9	13.8<br>14.0<br>14.3	24.5<br>25.3<br>26.2	1.6<br>1.6<br>1.6	Leaf litter	April 20th & May 3rd
		B	10.6602532 -61.3261689	Min<br>Mean<br>Max	62.0<br>65.0<br>68.0	12.0<br>12.0<br>12.0	0.0<br>0.0<br>0.0	53.2<br>57.9<br>62.7	468.9<br>5576.2<br>16298.3	13.3<br>14.0<br>14.8	24.7<br>25.3<br>25.8	1.6<br>1.9<br>2.2	Leaf litter	
		C	10.6605097 -61.326418	Min<br>Mean<br>Max	58.0<br>59.0<br>60.0	8.0<br>8.0<br>8.0	0.0<br>0.2<br>0.3	49.4<br>50.9<br>52.4	1217.6<br>3723.4<br>4691.7	14.1<br>14.3<br>14.6	24.4<br>25.1<br>25.8	1.1<br>1.5<br>1.8	Pebbles	

d.

River	Site	Loc	Latitude Longitude	Values	Depth (cm)	Width (m)	Flow (m/s)	Canopy cover (%)	Light intensity (lux)	DO (mg/L)	Temperature (°C)	Turbidity (NTU)	Sediment type	Date visited
Arima	1	A	10.692835 -61.292449	Min	45.0	12.0	0.0	4.1	3399.1	12.3	24.0	4.4	Silt	April 26th & May 10th
				Mean	68.5	12.0	0.0	9.5	16538.6	14.1	25.9	4.5		
				Max	92.0	12.0	0.0	14.9	29420.0	16.0	29.5	4.6		
		B	10.692755 -61.292442	Min	53.0	10.0	0.0	23.5	7129.7	12.9	23.9	1.3	Silt	
				Mean	54.5	10.0	0.0	25.1	23872.5	14.8	26.0	3.1		
				Max	56.0	10.0	0.0	26.6	56453.5	16.7	29.0	5.0		
		C	10.6927453 -61.292564	Min	50.0	12.0	0.0	1.4	4480.8	13.2	24.2	1.7	Silt	
				Mean	58.5	12.0	0.0	2.4	16661.6	15.0	25.4	3.8		
				Max	67.0	12.0	0.0	3.3	36184.9	16.8	26.8	5.8		
	2	A	10.6898261 -61.2902994	Min	51.0	2.0	0.0	51.3	2559.1	13.2	24.4	1.4	Pebbles	April 27th & May 10th
				Mean	55.0	2.0	0.2	72.8	5797.2	13.9	25.0	1.8		
				Max	59.0	2.0	0.4	94.3	13905.5	14.7	26.0	2.3		
		B	10.6910773 -61.2908238	Min	52.0	10.0	0.0	15.7	6829.7	13.0	24.3	1.5	Pebbles	
				Mean	53.0	10.0	0.0	22.6	33006.3	13.1	25.8	2.1		
				Max	54.0	10.0	0.0	29.5	50568.0	13.2	27.0	2.7		
		C	10.6910499 -61.2907148	Min	56.0	10.0	0.0	23.5	7463.2	12.6	24.2	1.5	Pebbles	
				Mean	57.0	10.0	0.0	26.7	22764.9	13.4	25.3	2.2		
				Max	58.0	10.0	0.0	29.9	35644.5	14.3	26.3	2.9		
	3	A	10.653088 -61.2831289	Min	51.0	3.0	0.2	26.8	1899.8	14.3	25.0	6.9	Sand	April 27th & May 1st
				Mean	54.0	3.0	0.3	28.4	8070.8	14.5	26.2	9.1		
				Max	60.0	3.0	0.4	31.6	18402.5	14.9	27.8	13.3		
		B	10.6534538 -61.2834823	Min	45.0	5.0	0.5	64.4	196.9	13.6	24.8	9.7	Sand	
				Mean	48.3	5.0	0.6	65.7	2423.7	14.0	26.0	10.3		
				Max	50.0	5.0	0.6	68.2	5033.5	14.8	27.4	11.6		
		C	10.6533681 -61.2835612	Min	53.0	2.0	0.0	44.0	791.3	14.8	25.6	9.0	Silt	
				Mean	58.3	2.0	0.0	44.0	12463.8	14.9	26.5	9.9		
				Max	61.0	2.0	0.0	44.0	24523.8	15.0	27.4	11.8		

e.

River	Site	Loc	Latitude Longitude	Values	Depth (cm)	Width (m)	Flow (m/s)	Canopy cover (%)	Light intensity (lux)	DO (mg/L)	Temperature (°C)	Turbidity (NTU)	Sediment type	Date visited
Guanapo	1	A	10.6956507 -61.2615404	Min	55.0	3.0	0.0	50.0	2770.5	13.1	24.4	1.5	Pebbles	April 28th & May 6th
				Mean	62.5	3.7	0.6	50.6	7640.1	13.8	24.7	1.6		
				Max	71.0	4.0	0.9	51.1	13297.7	14.4	24.9	1.6		
		B	10.6960796 -61.2617315	Min	61.0	4.0	0.7	0.0	6590.3	13.1	24.8	1.2	Rock	
				Mean	64.0	6.2	0.9	0.0	14411.9	13.9	25.0	1.4		
				Max	71.0	7.0	0.9	0.0	23191.9	14.6	25.4	1.7		
		C	10.6965746 -61.262246	Min	53.0	3.0	0.0	53.9	2489.9	13.3	24.4	1.0	Pebbles	
				Mean	62.5	4.7	0.0	55.0	20011.2	13.5	24.9	1.3		
				Max	69.0	6.0	0.1	56.0	40121.7	13.8	25.7	1.8		
	2	A	10.6932231 -61.2610646	Min	68.0	10.0	0.0	55.8	228.1	13.8	25.1	1.3	Pebbles	April 13th & May 6th
				Mean	79.5	10.2	0.1	59.6	1029.8	14.4	26.0	1.4		
				Max	91.0	10.5	0.2	63.3	2008.9	15.0	26.7	1.5		
		B	10.6935391 -61.2621615	Min	47.0	7.0	0.0	55.9	4015.7	14.0	25.3	1.6	Pebbles	
				Mean	64.0	8.5	0.2	56.8	5452.1	14.8	25.8	2.1		
				Max	81.0	10.0	0.4	57.8	9123.1	15.6	26.6	2.7		
		C	10.6940758 -61.2633045	Min	49.0	7.0	0.0	0.9	14754.2	14.0	25.6	1.5	Rock	
				Mean	75.5	8.7	0.2	2.4	25307.4	14.5	26.1	2.1		
				Max	102.0	10.5	0.4	3.8	35592.2	15.0	26.7	2.8		

f.

River	Site	Loc	Latitude Longitude	Values	Depth (cm)	Width (m)	Flow (m/s)	Canopy cover (%)	Light intensity (lux)	DO (mg/L)	Temperature (°C)	Turbidity (NTU)	Sediment type	Date visited
Aripo	1	A	10.6851116 -61.2324062	Min	40.0	5.0	0.0	56.8	150.6	10.4	24.2	1.2	Leaf litter	April 22nd & April 30th
				Mean	42.5	5.0	0.0	63.6	1000.3	11.0	24.6	3.2		
				Max	45.0	5.0	0.0	70.4	2794.9	11.7	24.9	5.1		
		B	10.6850535 -61.2324939	Min	55.0	4.0	0.0	57.1	111.6	10.4	24.2	1.2	Leaf litter	
				Mean	55.0	4.0	0.0	57.2	767.1	11.0	24.4	3.2		
				Max	55.0	4.0	0.0	57.2	1396.9	11.7	24.8	5.1		
		C	10.6857708 -61.2324635	Min	32.0	2.0	0.0	55.5	993.9	10.4	24.1	0.9	Pebbles	
				Mean	36.5	2.0	0.0	59.6	3996.7	11.4	24.6	1.8		
				Max	41.0	2.0	0.0	63.7	10404.8	11.9	25.1	2.8		
	2	A	10.6812097 -61.2302274	Min	79.0	5.0	0.0	92.7	2279.7	12.9	24.4	1.6	Leaf litter	April 22nd & April 30th
				Mean	79.0	5.0	0.0	94.4	4073.2	13.2	25.1	2.6		
				Max	79.0	5.0	0.0	96.1	5817.9	13.6	26.0	3.5		
		B	10.6814853 -61.2303903	Min	59.0	5.0	0.0	46.0	3850.8	11.9	24.6	1.9	Leaf litter	
				Mean	59.5	5.0	0.0	46.0	10951.9	12.7	25.3	7.7		
				Max	60.0	5.0	0.0	46.0	21479.1	13.6	25.9	13.5		
		C	10.6815743 -61.2305072	Min	66.0	3.0	0.0	54.2	614.6	11.9	24.4	1.1	Pebbles	
				Mean	66.0	3.0	0.0	54.2	5773.0	12.7	24.9	1.9		
				Max	66.0	3.0	0.0	54.2	12436.7	13.5	25.6	2.7		
	3	A	10.650799 -61.2225565	Min	55.0	5.0	0.0	55.8	1089.5	8.9	26.2	3.8	Leaf litter	April 25th & May 9th
				Mean	57.5	5.0	0.0	58.7	6944.6	11.2	26.7	3.9		
				Max	60.0	5.0	0.0	61.7	21076.7	13.5	26.9	4.0		
		B	10.656661 -61.222766	Min	50.0	8.0	0.0	54.9	555.1	9.6	26.3	2.6	Leaf litter	
				Mean	51.5	8.0	0.0	61.3	5257.3	11.6	26.8	3.9		
				Max	53.0	8.0	0.0	67.8	10138.1	13.7	27.2	5.3		
		C	10.6505221 -61.2230803	Min	53.0	12.0	0.0	0.0	11533.1	8.8	26.7	3.0	Pebbles	
				Mean	61.5	12.0	0.0	5.7	25921.8	11.4	27.4	5.1		
				Max	70.0	12.0	0.0	11.4	48829.7	14.0	27.8	7.2		

g.

River	Site	Loc	Latitude Longitude	Values	Depth (cm)	Width (m)	Flow (m/s)	Canopy cover (%)	Light intensity (lux)	DO (mg/L)	Temperature (°C)	Turbidity (NTU)	Sediment type	Date visited
Turure	1	A	10.6800449 -61.166981	Min	91.0	3.0	0.0	56.3	981.6	11.9	24.2	2.5	Sand	April 12th & May 8th
				Mean	92.0	3.0	0.0	57.8	11992.0	13.9	25.3	2.6		
				Max	93.0	3.0	0.0	59.4	36821.5	16.0	26.2	2.6		
		B	10.6802927 -61.1670844	Min	50.0	7.0	0.0	50.6	866.6	13.3	24.4	2.4	Leaf litter	
				Mean	57.5	7.0	0.0	51.5	1019.8	13.6	25.0	4.3		
				Max	65.0	7.0	0.0	52.3	1208.1	14.0	25.4	6.3		
		C	10.6806674 -61.1672407	Min	81.0	3.0	0.0	28.4	2676.3	11.6	24.6	1.6	Silt	
				Mean	84.0	3.0	0.0	46.6	3784.5	12.4	25.0	2.0		
				Max	87.0	3.0	0.0	64.9	5589.7	13.3	25.3	2.5		
	2	A	10.6571704 -61.1675028	Min	45.0	4.0	0.1	59.6	769.7	13.8	24.4	1.3	Leaf litter	April 24th & May 1st
				Mean	48.0	4.0	0.3	62.7	2103.6	14.1	25.8	1.4		
				Max	51.0	4.0	0.4	65.7	4291.0	14.5	27.2	1.5		
		B	10.657777 -61.1674847	Min	55.0	8.0	0.0	72.8	1051.5	14.0	24.2	0.9	Leaf litter	
				Mean	55.5	8.0	0.2	73.0	1723.3	14.4	25.6	1.3		
				Max	56.0	8.0	0.5	73.1	3210.0	14.8	26.9	1.7		
		C	10.6580249 -61.1675948	Min	57.0	5.0	0.0	47.5	5694.5	13.5	24.3	1.4	Leaf litter	
				Mean	58.0	5.0	0.0	47.5	12673.7	14.0	26.0	2.0		
				Max	59.0	5.0	0.0	47.5	30561.1	14.6	27.2	2.6		

Supplementary Table 2. Principal Component Analysis loadings where each variable (transformed as detailed in the second column) is loaded onto principal component axes. The last three rows report the summary of eigenvalues (standard deviation for each component). In bold are highlighted the variables with greater contribution to that principal component, and the components with eigenvalues >1. If all variables contributed equally to an individual principal component, we would expect that the sum of all square loadings divided by the number of principal components would equal 0.353, therefore any loading >0.353 (negative or positive) is considered to contribute more to a particular component.

Variable	Transformed	**PC1**	**PC2**	**PC3**	PC4	PC5	PC6	PC7	PC8
Canopy cover	Log	**-0.546**	0.045	-0.108	-0.081	0.554	-0.012	0.114	-0.601
Light intensity	Log	**0.508**	0.069	0.310	0.024	0.004	0.420	0.538	-0.418
Width	None	**0.436**	0.096	-0.256	-0.490	-0.138	-0.501	-0.197	-0.432
Flow rate	Log	0.126	**-0.720**	0.108	0.304	-0.037	0.120	-0.470	-0.354
Temperature	Log	0.302	**0.443**	-0.219	0.079	0.396	0.453	-0.543	0.041
Dissolved O_2	Log	0.324	**-0.438**	-0.126	-0.191	0.659	-0.147	0.232	0.375
Turbidity	Log	0.100	-0.030	**-0.781**	0.517	-0.131	-0.030	0.295	-0.086
Depth	None	0.178	0.278	**0.375**	0.593	0.254	-0.575	-0.038	-0.050
Standard deviation		***1.345***	***1.128***	***1.033***	*0.972*	*0.961*	*0.953*	*0.815*	*0.637*
Proportion of Variance		*0.226*	*0.159*	*0.133*	*0.118*	*0.115*	*0.113*	*0.083*	*0.050*
Cumulative Proportion		*0.226*	*0.385*	*0.518*	*0.636*	*0.752*	*0.865*	*0.949*	*1.00*

Supplementary Table 3. Model structure and output for the five behavioural metrics in response to guppy total length. The statistics were calculated using the drop1 function in 'stats' v. 4.3.0 package in R. Df refers to the test degrees of freedom as one term is removed from the model. The effect size **d** was calculated as the estimate for the fixed effect divided by the square root of the sum of variances of random effects (Brysbaert and Stevens, 2018). LRT is the Likelihood Ratio Test which measures the difference in fit between the full model and the reduced model. Pr(>Chi) represents the p-value associated with the likelihood ratio test statistic.

**Behavioural Response**	**Formula**	**Df**	**d**	**LRT**	**Pr(>Chi)**
**Presence/Absence**	Guppy length + (1| River/Location Code), family = binomial	1	0.016	0.256	0.612
**Time of First Visit**	Guppy length + (1| River/Location Code), family = nbinomi2	1	0.016	0.041	0.838
**Number of Visits**	Guppy length + (1| River/Location Code), family = nbinomi2	1	0.103	2.492	0.113
**Time Spent Near Stimulus**	Guppy length + (1| River/Location Code), family = nbinomi2, ziformula=~1	1	0.059	1.963	0.161
**Number of Attacks**	Guppy length + (1| River/Location Code), family = nbinomi2	1	0.058	1.880	0.170

Supplementary Table 4. Associations between PCs and presence/absence of each predator species based on the GLLVM. The rows in bold highlight significant interactions where the p-value < 0.05.

	Coefficients predictors	Estimate	Standard Error	z value	Pr(>|z|)
**PC1**	*Andinoacara pulcher*	0.134	0.099	1.356	0.1751
	Saxatilia frenata	0.063	0.097	0.646	0.5184
	Hoplias malabaricus	0.006	0.154	0.040	0.9677
	Hemibrycon taeniurus	0.183	0.099	1.834	0.0666 ·
	Anablepsoides hartii	**-0.751**	**0.207**	**-3.627**	**0.0002** ***
	Astyanax bimaculatus	-0.119	0.105	-1.137	0.2557
	Rhamdia cf. quelen	-0.074	0.175	-0.425	0.6708
	Roeboides dientonito	-0.117	0.171	-0.685	0.4932
**PC2**	*Andinoacara pulcher*	0.164	0.117	1.398	0.1620
	Saxatilia frenata	**0.336**	**0.121**	**2.783**	**0.0053** **
	Hoplias malabaricus	**0.585**	**0.226**	**2.584**	**0.0097** **
	Hemibrycon taeniurus	0.024	0.115	0.207	0.8359
	Anablepsoides hartii	0.027	0.175	0.155	0.8764
	Astyanax bimaculatus	-0.004	0.116	-0.039	0.9688
	Rhamdia cf. quelen	-0.217	0.159	-1.361	0.1735
	Roeboides dientonito	**0.576**	**0.263**	**2.189**	**0.0285** *
**PC3**	*Andinoacara pulcher*	-0.067	0.124	-0.547	0.5841
	Saxatilia frenata	0.066	0.123	0.539	0.5899
	Hoplias malabaricus	-0.207	0.183	-1.126	0.2601
	Hemibrycon taeniurus	0.061	0.127	0.479	0.6318
	Anablepsoides hartii	-0.168	0.170	-0.987	0.3235
	Astyanax bimaculatus	0.192	0.128	1.493	0.1354
	Rhamdia cf. quelen	**0.539**	**0.275**	**1.960**	**0.0499** *
	Roeboides dientonito	**-0.442**	**0.191**	**-2.312**	**0.0207** *

Supplementary Table 5. Full list of all models for the behavioural analysis. The details of the models including the random effects and error distribution are specified below each response variable (Y). For each response variable (a to e), a different set of models have been run on the entire dataset (Full Data) and for each treatment separately (Prey Data and Control Data). Each row represents a model where a different explanatory variable (X) is included and compared to a null model which only includes the random effects. ΔAICc: Difference in the small sample corrected AIC between each model and the most likely model (models are ordered by the ΔAICc). df: number of components for each model. In red are highlighted the null models' scores used in the comparison.

a.

**Presence/Absence**<br>glmmTMB(*Y* ~ *X* + (1| River/Location Code), family = binomial)								
**Full Data**	**ΔAICc**	**df**	**Prey Data†**	**ΔAICc**	**df**	**Control Data**	**ΔAICc**	**df**
PC2*PC3	0	6	PC2*PC3	0	5	PC3	0	4
PC2+PC3	1.2	5	PC2+PC3	1.6	4	PC2+PC3	0.8	5
PC3	3.6	4	PC3	3.7	3	PC2*PC3	2	6
PC2	5.6	4	PC2	5	3	PC1+PC3	2.2	5
PC1+PC3	5.6	5	PC1+PC3	5.8	4	*Null*	*3.3*	*3*
PC1*PC3	7.2	6	Control first	6.6	3	PC2	4.1	4
Null	*7.4*	*3*	*Null*	*6.9*	*2*	PC1*PC3	4.1	6
Time of day	7.4	4	Time of day	7	3	Time of day	4.9	4
PC1+PC2	7.5	5	PC1+PC2	7.1	4	Replicate	5.4	4
Treatment	8.6	4	PC1*PC3	8	5	Control first	5.4	4
Control first	8.6	4	Replicate	8.4	3	PC1	5.5	4
Replicate	9.4	4	PC1	8.7	3	PC1+PC2	6.2	5
PC1	9.5	4	PC1*PC2	9.2	5	PC1*PC2	8.3	6
PC1*PC2	9.7	6						
† These models included the random effect of river only: glmmTMB(*Y* ~ *X* + (1| River), family = binomial)								

b.

**Time of First Visit**<br>glmmTMB(*Y* ~ *X* + (1| River/Location Code), family = nbinom2)								
**Full Data**	**ΔAICc**	**df**	**Prey Data**	**ΔAICc**	**df**	**Control Data**	**ΔAICc**	**df**
PC2+PC3	0	6	PC2+PC3	0	6	PC2+PC3	0	6
PC2*PC3	2.1	7	PC2	0.9	5	PC3	0.9	5
PC2	2.5	5	PC2*PC3	1.7	7	PC2*PC3	1.3	7
PC1+PC2	4.3	6	PC1*PC2	3	7	PC1+PC3	2.7	6
PC1*PC2	4.8	7	PC1+PC2	3.1	6	PC1*PC3	5	7
PC3	11.5	5	Time of day	9.1	5	PC2	5.9	5
Time of day	11.6	5	PC3	9.8	5	*Null*	*6.4*	*4*
Replicate	12.1	5	Replicate	10	5	PC1+PC2	7.1	6
Null	*13*	*4*	*Null*	*10.5*	4	PC1	8.2	5
PC1+PC3	13.6	6	Control first	11.6	5	Replicate	8.3	5
Treatment	14.9	5	PC1+PC3	11.6	6	Time of day	8.3	5
PC1	15	5	PC1	12.2	5	Control first	8.4	5
Control first	15	5	PC1*PC3	13.8	7	PC1*PC2	8.5	7
PC1*PC3	15.4	7						

c.

**Number of Visits**<br>glmmTMB(*Y* ~ *X* + (1| River/Location Code), family = nbinom2)								
**Full Data**	**ΔAICc**	**df**	**Prey Data**	**ΔAICc**	**df**	**Control Data**	**ΔAICc**	**df**
PC2	0	5	PC2+PC3	0	6	PC2	0	5
PC2+PC3	0.5	6	PC2	0.4	5	PC1+PC2	1.7	6
PC2*PC3	1.4	7	PC2*PC3	1.1	7	PC2+PC3	2.1	6
PC1+PC2	1.7	6	PC1+PC2	2.3	6	PC2*PC3	3.8	7
PC1*PC2	3.8	7	PC1*PC2	4.1	7	PC1*PC2	3.9	7
Treatment	12	5	*Null*	*22.5*	*4*	Control first	6.7	5
Null	*20.7*	*4*	Replicate	23.5	5	*Null*	*7.1*	*4*
Replicate	21.4	5	PC3	23.8	5	Time of day	7.7	5
Time of day	21.9	5	Control first	24.3	5	Replicate	8.9	5
PC1	22.4	5	PC1*PC3	24.4	7	PC1	9.3	5
Control first	22.7	5	PC1	24.6	5	PC3	9.3	5
PC3	22.7	5	Time of day	24.7	5	PC1+PC3	11.5	6
PC1+PC3	24.4	6	PC1+PC3	25.9	6	PC1*PC3	13.8	7
PC1*PC3	25.5	7						

d.

**Time Spent Near Stimulus**<br>glmmTMB(*Y* ~ *X* + (1| River/Location Code), ziformula=~1, family = nbinom2)								
**Full Data**	**ΔAICc**	**df**	**Prey Data**	**ΔAICc**	**df**	**Control Data**	**ΔAICc**	**df**
Treatment	0	6	Replicate	0	6	Control first	0	6
PC2	36	6	PC2	0.4	6	*Null*	*0.9*	*5*
PC1+PC2	36.9	7	*Null*	*0.6*	*5*	Time of day	1.6	6
Replicate	37.3	6	PC1+PC2	1.9	7	PC2	2.3	6
Null	*37.3*	*5*	PC3+PC2	2.1	7	PC1	2.5	6
PC3+PC2	37.9	7	PC3	2.4	6	PC3	3	6
PC1*PC2	38.8	8	PC1	2.7	6	Replicate	3.1	6
PC2*PC3	39.1	8	Control first	2.7	6	PC2*PC3	3.2	8
PC1	39.2	6	Time of day	2.8	6	PC1+PC2	3.3	7
Time of day	39.4	6	PC2*PC3	4.1	8	PC3+PC2	4.5	7
PC3	39.4	6	PC1*PC2	4.1	8	PC1+PC3	4.8	7
Control first	39.4	6	PC1+PC3	4.6	7	PC1*PC2	5.5	8
PC1+PC3	41.3	7	PC1*PC3	6.2	8	PC1*PC3	7.1	8
PC1*PC3	43.1	8						

e.

**Number of Attacks**<br>glmmTMB(*Y* ~ *X* + (1| River/Location Code), family = nbinom2)								
**Full Data**	**ΔAICc**	**df**	**Prey Data**	**ΔAICc**	**df**	**Control Data**	**ΔAICc**	**df**
Treatment	0	5	PC2	0	5	PC1	0	5
PC2	89.2	5	PC2+PC3	1.7	6	*Null*	*0.1*	*4*
PC2+PC3	90.8	6	PC1+PC2	2.2	6	Time of day	0.4	5
PC1+PC2	91.3	6	PC1*PC2	3.6	7	Control first	0.9	5
PC1*PC2	92.8	7	PC2*PC3	3.8	7	Replicate	2	5
PC2*PC3	92.9	7	Time of day	4.7	5	PC1+PC3	2	6
Time of day	93.9	5	*Null*	*5.2*	*4*	PC1+PC2	2.2	6
Null	*94.1*	*4*	PC1	6	5	PC3	2.3	5
PC1	94.7	5	PC3	6.5	5	PC2	2.3	5
PC3	95.5	5	Replicate	6.7	5	PC1*PC3	3.2	7
Replicate	95.8	5	Control first	7	5	PC1*PC2	4	7
Control first	95.8	5	PC1+PC3	7.8	6	PC2+PC3	4.5	6
PC1+PC3	96.3	6	PC1*PC3	8.9	7	PC2*PC3	6.4	7
PC1*PC3	97.5	7						

Supplementary Figures

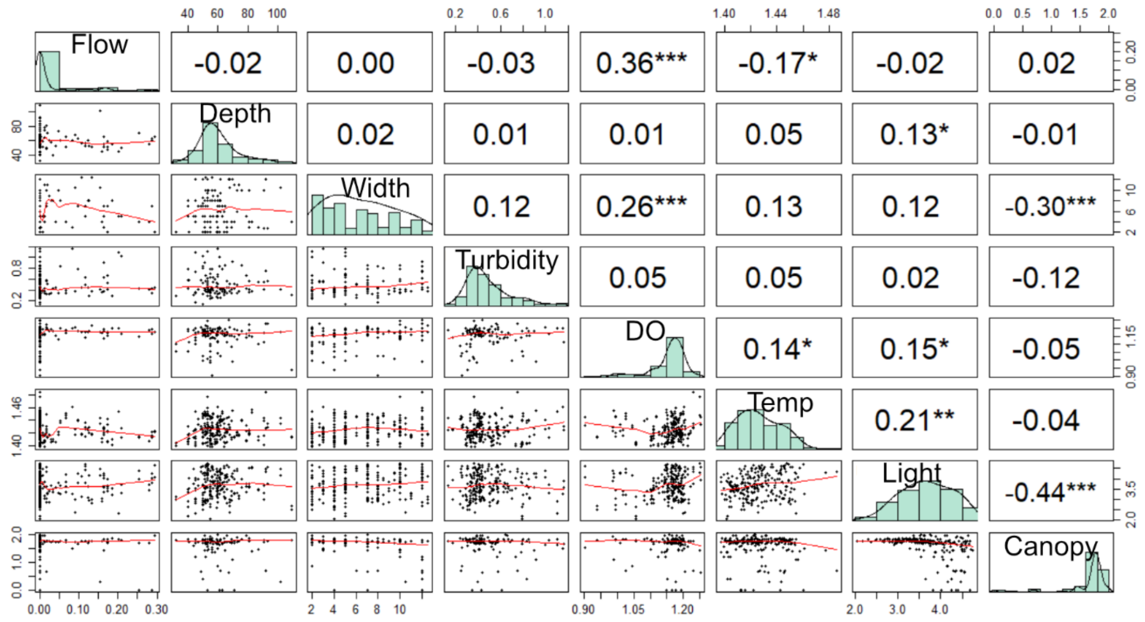

Supplementary Figure 1. Correlation matrix for all continuous environmental variables. There were significant positive correlations between temperature and time of day (Spearman's rank, $r_{(224)}=0.37$, $p<0.001$), flow rate and dissolved oxygen ($r_{(224)}=0.36$, $p<0.001$), dissolved oxygen and river width ($r_{(224)}=0.26$, $p<0.001$), and temperature and light intensity ($r_{(224)}=0.21$, $p=0.001$). Significant negative correlations were found between canopy cover and light intensity ($r_{(224)}= -0.44$, $p<0.001$), canopy cover and river width ($r_{(224)}= -0.30$, $p<0.001$), and time of day and light intensity ($r_{(224)}= -0.21$, $p<0.001$). The red lines represent the non-parametric estimate of the relationship between the variables.

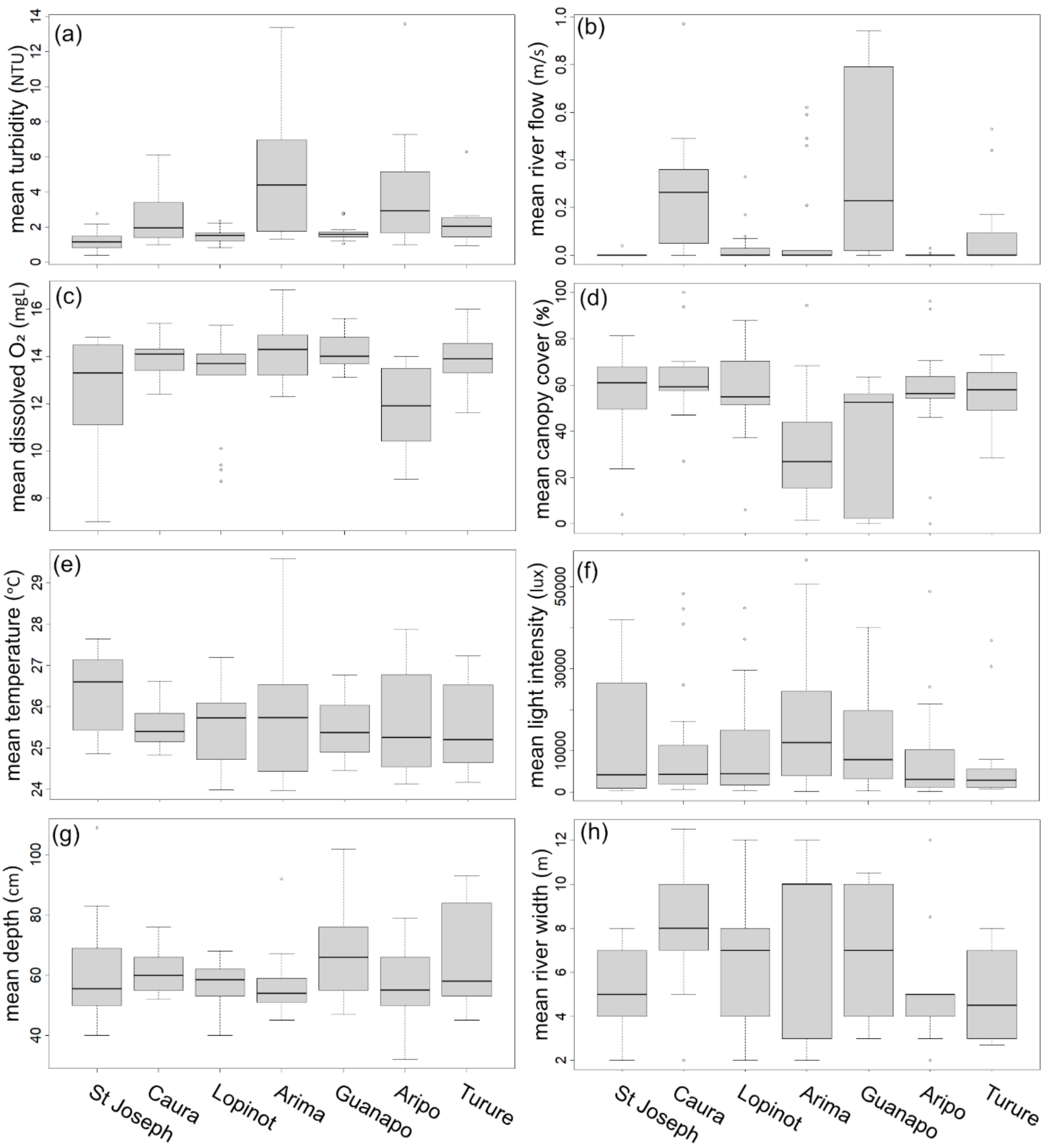

Supplementary Figure 2. Environmental parameters across rivers (N=228). There were significant differences between rivers regarding **(a)** turbidity levels (Kruskal-Wallis χ^2= 67.761, df = 6, p<0.001). A pairwise Dunn's test with Benjamini-Hochberg correction for multiple testing revealed significantly higher turbidity in the rivers Arima and Aripo compared to most other rivers (St Joseph, p_{Arima}<0.001 , p_{Aripo}<0.001; Caura, p_{Arima}=0.024, p_{Aripo}=0.185; Lopinot, p_{Arima}<0.001, p_{Aripo}<0.001 ; Arima, p_{Aripo}=0.308 ; Guanapo, p_{Arima}=0.001 , p_{Aripo}=0.015 ; Turure, p_{Arima}=0.017 , p_{Aripo}=0.127). **(b)** There were also differences in flow rates (χ^2= 83.033, df = 6, p-value <0.001), where the Caura, Guanapo and Turure had faster water flow (St Joseph, p_{Caura}<0.001 , $p_{Guanapo}$<0.001 , p_{Turure}=0.049 ; Caura, $p_{Guanapo}$=0.902 , p_{Turure}<0.001; Lopinot, p_{Caura}<0.001 , $p_{Guanapo}$<0.001 , p_{Turure}=0.593; Arima, p_{Caura}<0.001 , $p_{Guanapo}$=0.005 , p_{Turure}=0.986 ; Guanapo, p_{Turure}=0.013; Aripo, p_{Caura}<0.001, $p_{Guanapo}$<0.001, p_{Turure}=0.778). **(c)** Levels of dissolved oxygen also varied significantly across rivers (χ^2= 45.593, df = 6, p-value <0.001);

specifically, the Aripo and St Joseph had lower levels of dissolved oxygen (St Joseph, p_{Aripo}=0.029 ; Caura, p_{Aripo}<0.001,$p_{St\ Joseph}$=0.029 ; Lopinot, p_{Aripo}=0.049, $p_{St\ Joseph}$=0.385; Arima, p_{Aripo}<0.001, $p_{St\ Joseph}$=0.031; Guanapo, p_{Aripo}<0.001, $p_{St\ Joseph}$=0.014; Turure, p_{Aripo}=0.002, $p_{St\ Joseph}$=0.146). **(d)** There were also differences in the canopy cover across rivers (χ^2= 44.213, df = 6, p-value <0.001), with the Arima and Guanapo rivers with on average less vegetation cover (St Joseph, p_{Arima}<0.001, $p_{Guanapo}$=0.004; Caura, p_{Arima}<0.001, $p_{Guanapo}$=0.003; Lopinot, p_{Arima}=0.001, $p_{Guanapo}$=; Arima, $p_{Guanapo}$=0.476; Aripo, p_{Arima}=0.003, $p_{Guanapo}$=0.021; Turure, p_{Arima}=0.005, $p_{Guanapo}$=0.021). **(e)** Water temperature varied across rivers (χ^2= 16.562, df = 6, p-value= 0.011), with higher temperatures found in St Joseph river (Caura, p=0.016; Lopinot, p=0.016; Arima, p=0.032; Guanapo, p=0.016; Aripo, p=0.016; Turure, p=0.016). **(f)** Light intensity was also different (χ^2= 17.218, df = 6, p-value= 0.008); pairwise comparisons highlighted there were differences between Arima with Turure (p=0.012) and Aripo (p=0.012). **(g)** Depth was not found to differ between the majority of rivers except between Guanapo and Arima (χ^2=16.707, df = 6, p-value = 0.010, Dunn's Test p=0.021). Instead, width **(h)** varied across rivers (χ^2= 24.465, df = 6, p-value <0.001), particularly for Arima and Caura (St Joseph, p_{Arima}= 0.040, p_{Caura}=0.007; Caura, p_{Arima}= 0.570; Lopinot, p_{Arima}= 0.208, p_{Caura}=0.052; Guanapo, p_{Arima}= 0.699, p_{Caura}; Aripo, p_{Arima}=0.040, p_{Caura}=0.007; Turure, p_{Arima}=0.016, p_{Caura}=0.006). Horizontal black lines within the boxes represent the median value. The edges of the boxes represent the lower (25th percentile) and upper (75th percentile) quartiles. The whiskers extend from the most extreme data point by 1.5 × the interquartile range. The black circles represent outliers. There was a temperature gradient within the rivers (χ^2=17.719, df=2, p<0.001); pairwise comparisons using Dunn's test indicated that the most upstream sites were significantly cooler compared to the mid (p=0.003) and downstream (p<0.001) sites. Turbidity across sites did not follow any pattern with up, mid or downstream, and it was not related to quarry presence upstream (Mann-Whitney U-Test; W=7017.5, p=0.151). However, the proportion of sites with a silt substrate was higher than expected by chance for sites downstream from active quarrying (Chi-squared test; χ^2=7.75, df=1, p=0.005).

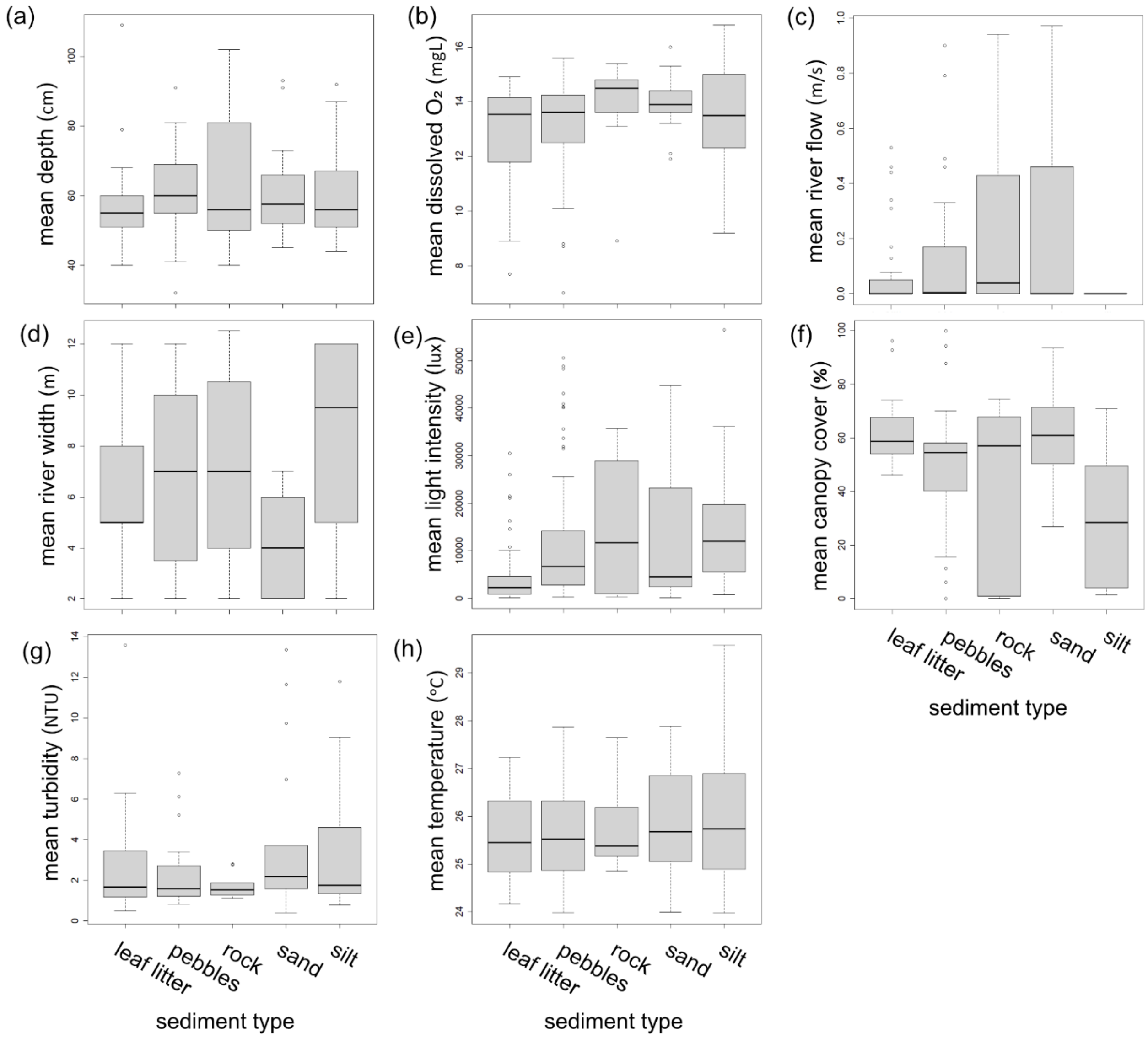

Supplementary Figure 3. Sediment type was associated with **(a)** depth (Kruskal-Wallis $\chi^2 = 9.9836$, df = 4, p=0.040), **(b)** dissolved oxygen ($\chi^2 = 13.996$, df = 4, p=0.007), **(c)** flow ($\chi^2 = 24.718$, df = 4, p<0.001), **(d)** river width (χ^2=22.531, df =4, p<0.001), **(e)** light intensity (χ^2= 39.152, df = 4, p<0.001) and **(f)** canopy cover (χ^2 =41.29, df =4, p<0.001). However it was not associated with **(g)** turbidity (χ^2 =7.3329, df = 4, p=0.1193) and **(h)** temperature (χ^2 = 2.4107, df = 4, p=0.6607). Horizontal black lines within the boxes represent the median value. The edges of the boxes represent the lower (25th percentile) and upper (75th percentile) quartiles. The whiskers extend from the most extreme data point by 1.5 × the interquartile range. The black circles represent outliers (N=228).

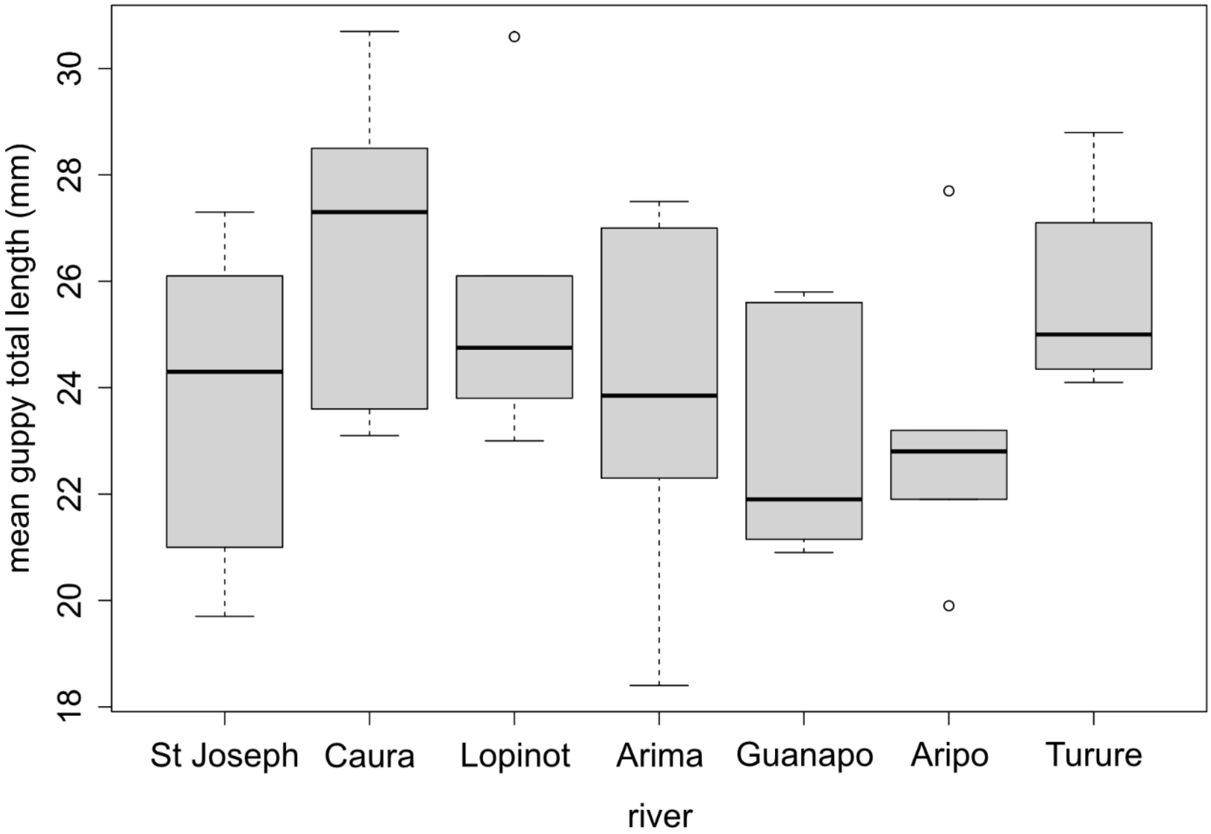

Supplementary Figure 4. Guppy size (total length) varied across rivers (N=361). Horizontal black lines within the boxes represent the median value. The edges of the boxes represent the lower (25th percentile) and upper (75th percentile) quartiles. The whiskers extend from the most extreme data point by 1.5 × the interquartile range. The black circles represent outliers.

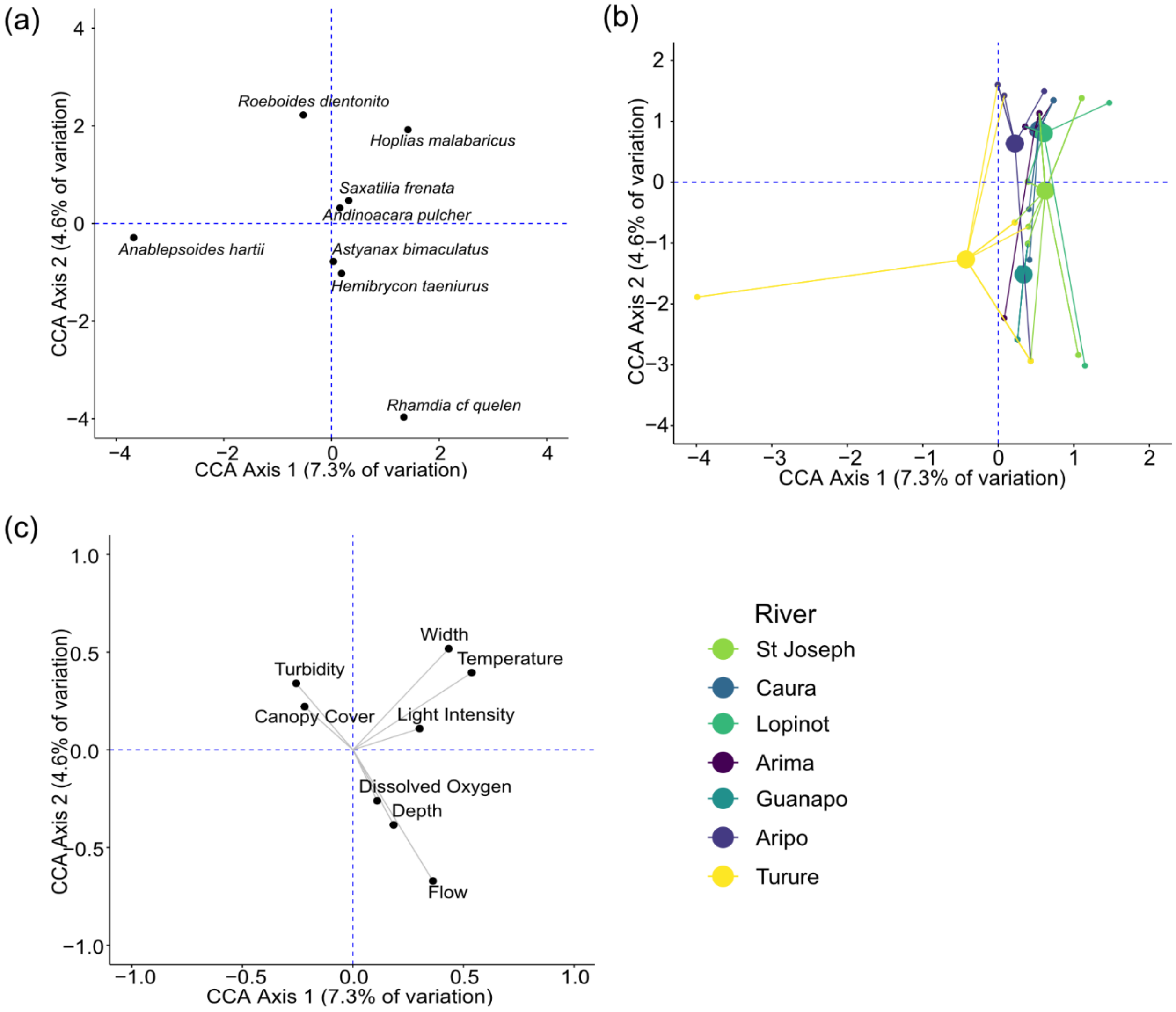

Supplementary Figure 5. Canonical Correspondence Analysis ordination of the observed predatory fish species and environmental variables across the seven rivers: **(a)** species, **(b)** sampling sites with lines representing the standard deviation of the mean of each river, and **(c)** all environmental variables.

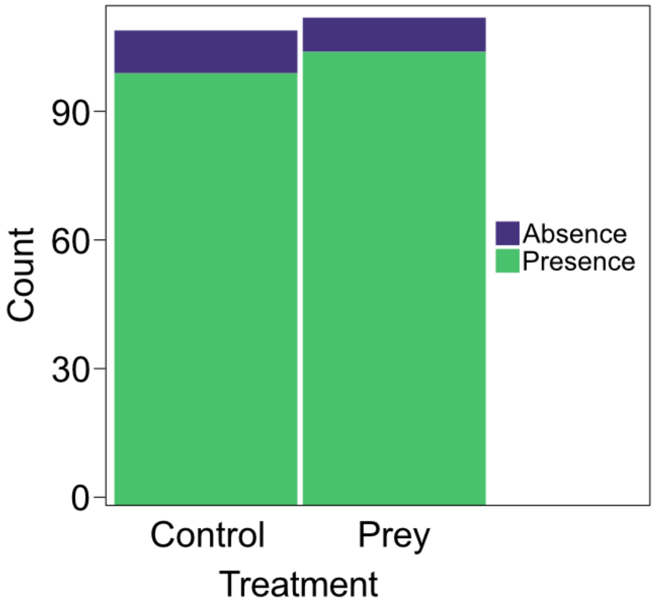

Supplementary Figure 6. Instances of recorded presence/absence for all predator species by treatment (i.e. control vs prey stimulus) (N=228).

Supplementary References

Brysbaert, M. and Stevens, M. (2018) ‘Power Analysis and Effect Size in Mixed Effects Models: A Tutorial’, *Journal of Cognition*, 1(1), p. 9.
