# Supplementary material for: Multiple environmental stressors affect predation pressure in a tropical freshwater system
Source: Commun Biol. 2024 May 29;7:663. doi: 10.1038/s42003-024-06364-6 (PMC11137014; doi:10.1038/s42003-024-06364-6)
Supplement: Supplementary file 3 — Description of additional supplementary files [file 42003_2024_6364_MOESM3_ESM.pdf]

Description of Additional Supplementary Files

File name: Supplementary Video

Description: Side-view videos:

clip #1: St Joseph site 2, circa 10:22 on 2nd May 2022 - Saxatilia frenata, Andinoacara pulcher and one Hoplias malabaricus performing attacks, Astyanax bimaculatus swimming past the apparatus

clip #2: Aripo site 3, circa 9:43 on 25th April 2022 - Andinoacara pulcher and Saxatilia frenata performing attacks, Hemibrycon taeniurus swimming past the apparatus

clip #3: Aripo site 3, circa 9:43 on 25th April 2022 - Roeboides dientonito performs one attack

clip #4: Turure site 2, circa 8:52 on 24th April 2022 - Andinoacara pulcher performs one attack
